# Rationale for and approach to preoperative opioid weaning: a preoperative optimization protocol

**DOI:** 10.1186/s13741-017-0079-y

**Published:** 2017-11-22

**Authors:** Heath McAnally

**Affiliations:** 1 Northern Anesthesia & Pain Medicine, LLC, 10928 Eagle River Rd #240, Eagle River, AK 99577 USA; 2 Department of Anesthesiology and Pain Medicine, University of Washington, Box 356540, Seattle, WA 98195-6540 USA

**Keywords:** Preoperative, Chronic pain, Opioid, Weaning, Optimization, Outcomes, Length of stay, Prehabilitation, Opioid-induced hyperalgesia, Biopsychosocial

## Abstract

The practice of chronic opioid prescription for chronic non-cancer pain has come under considerable scrutiny within the past several years as mounting evidence reveals a generally unfavorable risk to benefit ratio and the nation reels from the grim mortality statistics associated with the opioid epidemic. Patients struggling with chronic pain tend to use opioids and also seek out operative intervention for their complaints, which combination may be leading to increased postoperative “acute-on-chronic” pain and fueling worsened chronic pain and opioid dependence.

Besides worsened postoperative pain, a growing body of literature, reviewed herein, indicates that preoperative opioid use is associated with significantly worsened surgical outcomes, and severely increased financial drain on an already severely overburdened healthcare budget. Conversely, there is evidence that preoperative opioid reduction may result in substantial improvements in outcome. In the era of accountable care, efforts such as the Enhanced Recovery After Surgery (ERAS) protocol have been introduced in an attempt to standardize and facilitate evidence-based perioperative interventions to optimize surgical outcomes. We propose that addressing preoperative opioid reduction as part of a targeted optimization approach for chronic pain patients seeking surgery is not only logical but mandatory given the stakes involved. Simple opioid reduction/abstinence however is not likely to occur in the absence of provision of viable and palatable alternatives to managing pain, which will require a strong focus upon reducing pain catastrophization and bolstering self-efficacy and resilience.

In response to a call from our surgical community toward that end, we have developed a simple and easy-to-implement outpatient preoperative optimization program focusing on gentle opioid weaning/elimination as well as a few other high-yield areas of intervention, requiring a minimum of resources.

## Background

America’s opioid problem remains unrelenting, insidious, and without a clear solution in sight. One particular arena where aggressive intervention may have significant impact is the perioperative period. Within the surgical population, a large proportion of patients (as many as 33–70% (Tye et al. 2017)) seeking elective operations are already using chronic prescription opioids. These patients have been shown to demand greater doses and duration of opioid therapy postoperatively (VanDenKerkhof et al. 2012; Carroll et al. 2012; Hah et al. 2015; Rozet et al. 2014; Lawrence et al. 2008b; Armaghani et al. 2014). This may simply represent the presence of more serious “pain generators” leading to greater opioid consumption both pre- and postoperatively, or may represent a more complex scenario with compromised resilience and self-efficacy, increased underlying emotional and psychological distress, and frank opioid dependence driving the process. Chronic opioid use also reliably confers tolerance to the agent’s analgesic properties (Collett 1998; Adriaensen et al. 2003; Chang et al. 2007; Williams et al. 2013) leading to less effective perioperative pain management and often disproportionate pain. Furthermore, there is growing evidence that chronic opioid use increases pain sensitivity—currently labeled opioid-induced hyperalgesia (OIH)—thus leading to a pernicious cycle of increased demand for mu-receptor agonism/narcotization which in turn increases pain which increases demand (Angst and Clark 2006; Lee et al. 2011).

Seasoned surgeons of all disciplines have learned that the risk to benefit ratio of surgery may lie toward the harmful for elective procedures in patients who are morbidly obese, nutritionally deficient, deconditioned/“sarcopenic”, tobacco users, etc. A growing body of evidence (reviewed below) indicates that even after adjusting for many of these obvious confounders, chronic opioid use also predisposes to poor surgical outcomes including immediate postoperative complications such as infection and other physiologic perturbation (e.g., ileus and respiratory suppression with atelectasis and pneumonia) but also long-term issues such as compromised wound healing and reduced arthroplasty or intervertebral fusion success.

We propose that bringing opioid-dependent patients to the operating room for elective surgery carries an unacceptably high risk of conferring “acute-on-chronic pain” much like unaddressed hypovolemia in the chronic renal-insufficient patient invites acute-on-chronic renal failure. We also propose that among numerous high-yield targets for optimizing surgical outcome (e.g., tobacco cessation and nutritional prehabilitation), opioid reduction or elimination deserves particular consideration in the context of the growing public health problem of misuse, abuse, and dependence. Finally, we propose that deferring elective surgery until adequate preoperative optimization (i.e., reduction or elimination if possible) of opioid use, just like delaying for body mass index (BMI) improvements or tobacco cessation, comprises wise stewardship of private and public monies funding healthcare.

## Survey

**Fig. 1:**
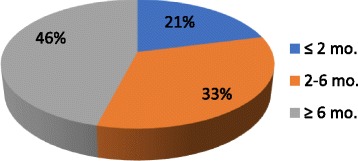
Acceptable preoperative optimization delay (by % of surgeons)

**Fig. 2:**
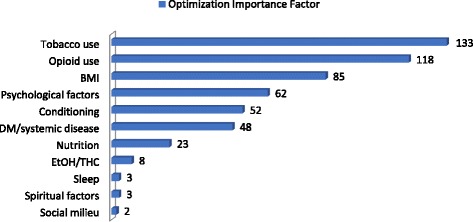
Preoperative variables of concern to surgeons

We surveyed 62 local surgeons (with 48 respondents) from the disciplines of general surgery, gynecology, neurosurgery, ophthalmology, orthopedics, otolaryngology, plastic surgery, and urology to see how long they would be willing to defer elective operations in unprepared patients, and also what the top three to five issues (modifiable risk factors) were they would like to see optimized prior to elective surgery. The results are shown in Figs. 1 and 2. The range of perceived acceptable preoperative delay for non-urgent problems ranged from 4 weeks to indefinite, with the majority of surgeons favoring waiting longer than 2–3 months if necessary to optimize their patients. Among the common modifiable risk factors that our surgeons prioritized for preoperative optimization, the most frequently identified issues were tobacco use, opioid use, obesity, unrealistic expectations and other psychological factors, deconditioning, and diabetes or other systemic diseases.

A weighted value (“optimization importance factor”) was created for each variable reported by our surgeon population by multiplying the number of surgeons reporting it by a numeric scale ranking (in descending order from 5 to 1) of prioritization.

## Literature review

Over the past 10 years, multiple reports (Armaghani et al. 2014; Lawrence et al. 2008a; Anderson et al. 2009; Roullet et al. 2009; Zywiel et al. 2011; Raebel et al. 2013; Pivec et al. 2014; Menendez et al. 2015; Morris et al. 2015; Hina et al. 2015; Aasvang et al. 2016; Morris et al. 2016; Sing et al. 2016; Zarling et al. 2016; Nguyen et al. 2016; Faour et al. 2017; Ben-Ari et al. 2017; Villavicencio et al. 2017; Waljee et al. 2017; Smith et al. 2017; Rozell et al. 2017; Chan et al. 2017; Cheah et al. 2017) have noted a generally consistent association between preoperative chronic opioid use and worsened postoperative outcomes. These studies are summarized in Table 1, and individual studies of interest are briefly presented below. A negative study is discussed first, however, along with criticism.

**Table 1:** Studies reporting outcomes following preoperative opioid use

Authors	Year	Discipline	Findings
Lawrence et al.	2008	Spine	47 chronic opioid-using patients experienced significantly worsened outcomes of pain and disability postoperatively compared to 44 non-opioid-using patients
Anderson et al.	2009	Spine	Logistical regression model of 488 ACDF patients revealed “weak” preoperative opioid use as an independently significant negative predictive variable for postoperative neck disability
Roullet et al.	2009	Orthopedics	12 chronic preoperative opioid-using patients had greater postoperative opioid consumption and phantom limb pain than 10 non-opioid-using controls
Zywiel et al.	2011	Orthopedics	45 TKA patients using opioids preoperatively had significantly greater postoperative dysfunction and complications including need for revision compared to non-opioid-using controls
Raebel et al.	2013	Bariatric surgery	77% of 933 chronic opioid-using bariatric surgery patients continued to use opioids at 12-month postoperative follow-up and generally at higher doses
Pivec et al.	2014	Orthopedics	54 THA patients using opioids preoperatively had significantly greater postoperative pain and opioid consumption, increased hospital LOS, worsened postoperative function and increased arthroplasty failure compared to non-opioid-using controls
Armaghani et al.	2014	Spine	Logistic regression model of 583 patients demonstrated that of 321 patients using chronic preoperative opioids, “increasing preoperative use” correlated with ongoing opioid dependence 12 months postoperatively
Kelly et al.	2015		762 “weak opioid” (codeine, propoxyphene, hydrocodone), 226 “strong opioid” (meperidine, morphine, oxycodone) and 16 non-opioid-using patients showed no significant differences in pain and disability at 2-year follow-up
Menendez et al.	2015	Orthopedics	15,901 THA, TKA, TSA, and spine fusion patients with a diagnosis of opioid dependence or abuse had statistically significantly worsened morbidity and mortality outcomes than over 9 million controls
Hina et al.	2015	Orthopedics	28 opioid-using orthopedic patients displayed significantly greater hyperalgesia preoperatively, reported greater pain and consumed significantly more opioids postoperatively than 40 non-opioid-using controls
Morris et al.	2015	Orthopedics	32 Reverse TSA patients using chronic preoperative opioids had significantly worse outcomes of postoperative shoulder function including ROM compared to 36 controls
Morris et al.	2016	Orthopedics	60 TSA patients using chronic preoperative opioids had significantly worse outcomes of postoperative shoulder function including ROM and decreased satisfaction compared to 164 controls
Sing et al.	2016	Orthopedics	116 TKA and THA patients using preoperative opioids had significantly worse postoperative pain and increased opioid consumption, increased hospital LOS, admission to rehabilitation facilities, worsened surgical outcomes (including arthrofibrosis and periprosthetic fractures) compared to 58 non-opioid-using controls
Aasvang et al.	2016	Orthopedics	58 TKA patients using chronic preoperative opioids had significantly increased pain and opioid requirements within the first week of surgery compared to 57 non-opioid-using controls
Zarling et al.	2016	Orthopedics	106 TKA and THA using chronic preoperative opioids had greater likelihood of postoperative rehabilitation facility admission and significantly increased continued use of opioids 12 months postoperatively than 209 non-opioid-using controls
Nguyen et al.	2016	Orthopedics	
Faour et al.	2017	Spine	77 ACDF patients using chronic preoperative opioids had significantly lower incidence of return to work than 204 non-opioid-using controls
Tye et al.	2017	Spine	80 lumbar decompression patients from the worker’s compensation population using chronic preoperative opioids greater than 3 months were significantly less likely to return to work than those (*n* = 60) using opioids less than 3 months preoperatively
Ben-Ari et al.	2017	Orthopedics	12,772 TKA patients using chronic preoperative opioids had significantly higher incidence of revision than 19,864 non-opioid-using controls
Smith et al.	2017	Orthopedics	36 TKA patients using chronic preoperative opioids had significantly worse pain 6 months postoperatively compared to 120 non-opioid-using controls
Waljee et al.	2017	General surgery	17,577 patients using chronic preoperative opioids had significantly longer hospital LOS, increased incidence of discharge to rehabilitation facilities and hospital readmission, and generated significantly higher financial expenditures than 182,428 controls
Villavicencio et al.	2017	Spine	60 TLIF patients using chronic preoperative opioids had significantly greater 12-month postoperative pain and disability compared to 33 non-opioid-using controls
Rozell et al.	2017	Orthopedics	275 TKA and THA patients using chronic preoperative opioids (compared to 527 controls) were shown in a regression model to be more likely to require increase perioperative opioids and increased hospital LOS as well as suffer higher incidence of complications
Chan et al.	2017	Orthopedics	36 TKA patients maintained on methadone preoperatively required greater postoperative opioids and inpatient pain management consultation, and had increased hospital LOS compared to 36 matched controls
Cheah et al.	2017	Orthopedics	138 TSA patients using chronic preoperative opioids had significantly greater postoperative pain and opioid use compared to 124 non-opioid-using controls

Kelly et al.’s (2015) retrospective cohort study indicated no evident difference in pain or disability 2 years out from cervical disc replacement or interbody fusion patients stratified into those using “weak” (*n* = 762) vs. “strong” (*n* = 226) opioids. A third control arm comprised only 16 individuals who were opioid-naïve going into surgery. The opioid categorization however was arbitrary and did not accurately reflect potency (e.g., meperidine which is considerably less potent than hydromorphone was placed in the “strong opioid” group whereas hydrocodone, essentially equipotent to morphine, was placed in the “weak opioid” group). Furthermore, there was undoubtedly considerable overlap between the two groups in terms of actual equivalent opioid dosage. This study must then be interpreted with considerable skepticism.

Menendez et al. (2015) performed a tremendously powered retrospective analysis of 15,901 opioid-dependent individuals undergoing various arthroplasties or spinal fusion operations, compared to over 9 million controls. After adjusting for demographic, comorbidity, hospital, and operative variables, they noted that opioid dependence was statistically significantly associated with increased morbidity and mortality. Increased hospital length of stay (LOS) and discharge to rehabilitation facilities also correlated with opioid dependence.

A small but interesting study by Hina et al. (2015) adds to the literature on opioid-induced hyperalgesia and demonstrated that preoperative opioid use is associated with demonstrable OIH prior to operation, and despite a more aggressive intraoperative anesthetic approach (e.g., inclusion of ketamine into the anesthetic), patients using chronic preoperative opioids displayed greater postoperative pain and increased opioid demand, consistent with the experience of most clinicians and the literature.

Sing et al. (2016) in a subgroup analysis examined the negative consequences of preoperative extended-release/long-term opioid use in relation to postoperative outcomes. They reported that of 116 total knee and hip arthroplasty patients using preoperative opioids, there was a trend noted for more severe outcomes (increased postoperative pain and opioid consumption, increased hospital LOS, increased admission to rehabilitation facilities, worsened surgical outcomes including arthrofibrosis and periprosthetic fractures) in the extended-release/long-acting opioid-using subgroup compared to the immediate-release opioid-using group.

Ben-Ari et al. (2017) in another highly powered study demonstrated that 12,772 patients undergoing total knee arthroplasty who had been using chronic preoperative opioids were significantly more likely to undergo revision arthroplasty compared to 19,864 controls, results echoed by other studies as listed in Table 1.

A few studies have examined the associated between preoperative opioid use and economic outcomes. Waljee et al. (2017) performed a retrospective analysis of nearly 200,000 patients undergoing various general surgical operations and hysterectomies. The patients using chronic preoperative opioids (*n* = 17,577) had significantly longer hospital LOS, increased incidence of discharge to rehabilitation facilities and hospital readmission, and generated greater than double the financial costs than 182,428 controls, after adjusting for numerous psychosocial variables. Faour et al. (2017) and Tye et al. (2017) have demonstrated that cervical and lumbar spine surgical patients, respectively, who used chronic preoperative opioids had significantly lower incidence of return to work than matched cohorts who did not use chronic preoperative opioids.

Finally, a very interesting study by Nguyen et al. (2016) suggests that preoperative opioid reduction may prove protective against these negative outcomes. Forty-one patients who successfully weaned their opioid burden prior to surgery by at least 50% were compared to 41 opioid-dependent patients who did not, and 41 opioid-naïve controls. The intervention group had outcomes comparable to the opioid-naïve group, with both of those two groups demonstrating significantly improved pain and functional outcomes compared to the patient group that did not reduce their opioid use preoperatively. This is the first study to demonstrate efficacy of preoperative opioid reduction.

## Discussion

The International Association for the Study of Pain has labeled 2017 the Global Year Against Pain After Surgery (International Association for the Study of Pain 2017). This laudable, ambitious approach highlights well-accepted and traditional objectives such as aggressive perioperative multimodal analgesia, and its goals are echoed in the philosophy of constructs such as Toronto’s Transitional Pain Service and the American Society of Anesthesiologists’ Perioperative Surgical Home (Katz et al. 2015; Desebbe et al. 2016). As important as such diversification of response is, it still comprises response and is thus at least theoretically inferior to an approach of proactive “pain prehabilitation,” certainly within the chronic pain patient population.

### The biologic complexity of chronic pain, and opioid-induced hyperalgesia

Pain is a complex and subjective experience now well-known to occur outside of the context of nociception, and with considerable potential for both amplification and suppression from the central nervous system (Latremoliere and Woolf 2009; Tracey and Mantyh 2007; Heinricher et al. 2009). While acute/nociceptive pain comprises a warning system alerting the organism to adopt avoidant behavior, it is now widely accepted (and supported by functional imaging evidence) that chronic pain for the most part represents maladaptive neuroplastic changes at the dorsal horn, and multiple higher (brain) centers including amygdala, hippocampus, insula, cingulate, and other parietal cortex areas and the prefrontal cortex (Flor et al. 1997; Tinazzi et al. 1998; Apkarian et al. 2004; Apkarian et al. 2005; Schmidt-Wilcke 2008; Rodriguez-Raecke et al. 2009; Baliki et al. 2010; Malinen et al. 2010; Apkarian et al. 2011; Farmer et al. 2012; Baliki et al. 2012).

Chronic opioid use is believed to reinforce this pathology, and again, functional neuroimaging studies seem to support shared neuroanatomic pathways and perturbances (Wanigasekera et al. 2011; Younger et al. 2011).

Chronic opioid use has also been shown to increase pain sensitivity via a process known as opioid-induced hyperalgesia (OIH). Distinct from tolerance, which represents an increasing threshold for analgesic responsiveness, OIH represents a reduced threshold for pain perception. Teleologically, the prolonged and imbalanced exogenous suppression of pain impulses to the brain should result in a reactionary increase in sensitivity to maintain homeostasis of this most critical protective sense. OIH is likely multifactorial with a host of postulated mechanisms including structural and functional alterations in opioid receptors, long-term potentiation at the dorsal horn, glial-mediated neuroinflammation, enhanced descending pain facilitation, and epigenetic factors (Lee et al. 2011; Roeckel et al. 2016; Weber et al. 2017). Once thought to require months of exposure to opioids, it is now recognized from animal and human investigations that OIH may occur with exposure as brief as days (Angst and Clark 2006; Compton et al. 2003; Cooper et al. 2012) and the recent widespread use of intraoperative remifentanil infusions has demonstrated that exposure on the order of hours is sufficient (Guignard et al. 2000; Angst et al. 2003). There may be a dose-response effect (Salengros et al. 2010; Fechner et al. 2013; Fletcher and Martinez 2014; Mauermann et al. 2016).

A specific association between chronic preoperative opioid use and postoperative hyperalgesia has been demonstrated recently (Hina et al. 2015; Chapman et al. 2011). This may result from preoperative OIH persisting into the postoperative period or may be mediated more acutely by requisite increased intra- and postoperative opioid doses. The association may also be confounded in the perioperative setting by inadequate analgesia resulting from tolerance; increased immediate postoperative pain intensity has been shown to correlate with increased incidence of persistent postoperative hyperalgesia (Malik et al. 2017; Weinbroum 2017).

Regardless of the mechanisms involved, the study by Nguyen et al. (2016) showing improved pain and functional outcomes after even a 50% reduction in preoperative opioid burden argues convincingly in the context of the other literature reviewed herein for a concerted effort toward preoperative opioid reduction if not elimination. Additional supporting evidence for this tactic comes from the recent demonstration that downtitration of remifentanil infusion rates is associated with a lower incidence of OIH (Comelon et al. 2016).

### The psychosocial complexity of chronic pain and opioid misuse

Chronic pain is also associated with psychological distress such as anxiety, post-traumatic stress disorder, borderline personality disorders, and to a lesser degree depression (Fishbain et al. 1998; Von Korff et al. 2005; Gureje et al. 2008; Bushnell et al. 2013; Simons et al. 2014). The reported severity of such chronic pain has been shown to correlate much more closely with these psychosocial variables than with somatic contributors including injury severity, which has in fact been shown in several studies to be non-predictive (Harris et al. 2007; Jenewein et al. 2009; Trevino et al. 2013). These psychological/behavioral comorbidities have also been shown to predict chronic postoperative pain specifically (Kleiman et al. 2011; Theunissen et al. 2012; Attal et al. 2014; Hoofwijk et al. 2015).

The distinct construct of pain catastrophizing has received significant attention recently in the arenas of pain management and perioperative medicine. Pain catastrophizing is defined as persistent negative cognitive and affective responses to actual or anticipated pain (Quartana et al. 2009) and incorporates various degrees of magnification, rumination, and perception of helplessness (Sullivan et al. 1995). Pain catastrophizing and learned helplessness correlate with many of the underlying psychiatric comorbidities mentioned above and also independently confer worsened postoperative pain (Theunissen et al. 2012; Ip et al. 2009; Khan et al. 2011; Vissers et al. 2012; Denison et al. 2004) and surgical outcomes (Abbot et al. 2011, Coronado et al. 2015, Teunis et al. 2015)

All of these behavioral comorbidities are strongly associated with chronic opioid use, misuse, and dependence (Turk et al. 2008; Becker et al. 2008; Goldner et al. 2014; Gross et al. 2016; Arteta et al. 2016; McAnally 2017) and in fact have been shown repeatedly to be the most robust predictors (Martins et al. 2012; Katz et al. 2013; Blanco et al. 2013).

A final consideration related to the complex association of psychopathology, chronic pain, opioid use, and the perioperative arena is that patients suffering with chronic pain are more likely to seek not only opioid prescriptions, but also operative intervention. Among those patients struggling with chronic pain is a disproportionate number of individuals plagued with catastrophic thinking regarding pain, as well as poor self-efficacy. To quote Beth Darnall, a leading contemporary researcher in the field, “pain catastrophizing may speed the path to surgery while simultaneously undermining surgical response” (Darnall 2016). In other words, the path to the operating room may be disproportionately self-selected by the very people who are least likely to benefit from it, or who are the least prepared at any rate.

Over-eager desire for surgical intervention and persistent seeking of opioid prescriptions are both more likely to be associated with an external locus of control. While impossible to measure objectively, this lack of self-efficacy may in fact be the most important independent variable.

### The rationale for preoperative opioid cessation and an effective biopsychosocial substitute

An increasing number of publications as well as our local survey of surgeons indicate the importance of preoperative opioid reduction. The survey results may be biased somewhat in terms of the importance associated to preoperative opioid reduction or elimination given our reputation and that of our preoperative optimization program within the community. Nonetheless, the literature does support both plausibility and rationality of this objective, and at an anecdotal level, anyone involved in perioperative care for more than a handful of years has learned the challenges involved in rendering opioid-tolerant patients comfortable in the post-anesthesia care unit and the ward, and surgeons and pain physicians are well aware of the difficulties they face afterward. As discussed above, there is growing recognition also that chronic preoperative opioid use confers postoperative problems beyond simple analgesic compromise. However, answering the question at hand, whether preoperative opioid reduction/elimination is beneficial in terms of outcome may be more difficult than appears on the surface. First of all, randomization is almost certainly not going to occur—patients either are or are not willing to reduce or eliminate their opioids preoperatively. Second, blinding would be nearly impossible in that the high probability of withdrawal symptoms would likely unmask treatment arms. Whether or not preoperative opioid reduction is beneficial must then most likely be judged from non-randomized prospective or retrospective studies, the plausibility of compelling “reverse” evidence such as the studies discussed herein, and common sense given the known associations between chronic opioid use and its harms.

Beyond mere opioid reduction/cessation, in view of the complex risk factors for chronic pain discussed briefly above, it stands to reason (and has been advocated by numerous experts, consensus groups, and clinical practice guidelines) (Veterans Health Administration and Department of Defense 2002; Chou et al. 2007; Federation of State Medical Boards 2013; United States Department of Health and Human Services 2016; Manchikanti et al. 2017) that a biopsychosocial-spiritual paradigm with particular focus upon enhancing resilience and diversify coping skills is required. Simply removing opioids without providing effective substitute coping mechanisms will invariably lead to non-compliance and dropout. A systematic, rigorous (e.g., weekly visit) program of opioid reduction/withdrawal palliation needs to be coupled with basic preoperative counseling addressing the replacement of multifactorial “wellness-killers” (e.g., poor self-valuation and esteem, unaddressed psychopathology, poor sleep, poor nutrition, sedentary lifestyle, tobacco use) with proactive steps supporting personal responsibility for health and wellness.

Growing recognition of the disproportionate impact of chronic pain syndromes upon operative outcomes in Canada has led to the establishment of what appears to be a promising, comprehensive approach to perioperative pain management for chronic pain patients with the Toronto General Transitional Pain Service (TPS) (Katz et al. 2015). The current iteration of the TPS involves five anesthesiology-based pain physicians, a palliative care specialist/family physician, two clinical psychologists and trainees, three acute pain nurse practitioners, two physical therapists with expertise in acupuncture, an exercise physiologist, and administrative staff (Katz et al. 2015).

The American Society of Anesthesiologists, among other organizations, has championed the concept of a Perioperative Surgical Home (PSH) (Desebbe et al. 2016) which is intended to address multiple perioperative health deficits at an institutional level. One of the theoretical functions of a PSH would be to address perioperative chronic pain management optimization including opioid reduction (Vetter and Kain 2017). A significant practical barrier however in the USA (with payer source fragmentation and limitations of reimbursement allocation) is actually coming up with the resources for such an effort. As noted by Vetter and Kain,

How will an organization finance these additional resources necessary for a Transitional Pain Service? …A small community hospital may be hard-pressed to mobilize the comprehensive services and personnel required to successfully implement a full-scale perioperative Transitional Pain Service.

We propose that moving such perioperative chronic pain optimization functions outside of the institution to a smaller, leaner paradigm shaped by market pressures including outcomes-driven referral patterns will result in more efficient use of resources and improved care. Toward that end, we have created and begun implementation of a multidisciplinary preoperative optimization program for chronic pain patients focusing on a few high-yield areas of intervention, with opioid reduction and pain catastrophizing as two of the top priorities (as well as tobacco cessation and diet and activity improvements). The current iteration of the program comprises a 10–12-week course and incorporates traditional preoperative assessment and consultation issues (e.g., cardiac clearance and endocrinologic optimization) into a basic “wellness program” with simple, graded, measurable objectives including gentle opioid weaning along the lines of the fairly standard 10% per week paradigm (Manchikanti et al. 2017). Evidence from the behavioral world indicates that it takes at least 12 weeks to change habits (Lally et al. 2010); moving such “pain prehabilitation” into the outpatient realm and allowing for adequate optimization time beforehand allows for such and furthermore overcomes the institutional-level problem of lack of resources by placing this critical component of healthcare into the hands of invested providers. While the cost of a dozen or so outpatient follow-up visits may seem formidable up front, it is exceeded by the cost of a single extra day in the hospital and pales in comparison to a canceled operation, or worst of all an adverse outcome.

## Conclusions

The literature increasingly supports an association between preoperative opioid use and worsened postoperative pain, surgical outcomes, length of stay, and financial costs. Conversely, there is evidence that preoperative opioid reduction may result in substantial improvements in outcomes. In order to optimize chronic pain patients seeking surgery, we propose that addressing preoperative opioid reduction (among a handful of high-yield risk factors) is not only logical but also imperative. Simple opioid reduction/abstinence however is not likely to occur in the absence of provision of viable and palatable alternatives to managing pain, which will require a strong focus upon reducing pain catastrophization and bolstering self-efficacy and resilience, and nurturing a commitment to overall biopsychosocial-spiritual health. We have developed a simple and easy-to-implement outpatient preoperative optimization program focusing on gentle opioid weaning/elimination along with tobacco cessation, “de-catastrophization” and expectation management, and nutritional, sleep, and conditioning “prehabilitation.” This 3-month program requires a minimum of resources and promises a good return on investment for chronic pain patients willing to exert a nominal degree of effort toward improving their surgical experience and outcome.

## Abbreviations

BMI: Body mass index
ERAS: Enhanced Recovery After Surgery
LOS: Length of stay
OIH: Opioid-induced hyperalgesia
PSH: Perioperative Surgical Home
TPS: Transitional Pain Service
